# Patterning 2D materials for devices by mild lithography

**DOI:** 10.1039/d1ra04982h

**Published:** 2021-09-06

**Authors:** Marcel Weinhold, Peter J. Klar

**Affiliations:** Institute of Experimental Physics I and Center for Materials Research (ZfM), Justus Liebig University Giessen Heinrich-Buff-Ring 16 DE-35392 Giessen Germany peter.j.klar@physik.uni-giessen.de

## Abstract

2D materials have been intensively studied for almost two decades and are now exhibiting exceptional properties. Thus, devices that integrate 2D materials offer many novel functionalities that will contribute significantly to the transition into an era beyond ‘Moore’. Lithographic methods are key technologies in the context of materials' integration into devices. However, to fully leverage the capabilities of these potential devices, it is vital to keep the integrity of the 2D materials intact and to minimize damage induced by device processing. This requirement is only partially met when employing conventional lithography methods, as they induce structural defects in the delicate materials. We demonstrate that exposing graphene to typical electron doses used in conventional electron beam lithography induces significant defect formation. The defect density is proportional to the electron dose and the structural integrity cannot be fully recovered by thermal annealing. We introduce a novel approach of mild lithography which combines traditional processing methods with a subsequent transfer step of the patterned mask onto the 2D material. We demonstrate that this separation of pattern definition and pattern application allows the lithographic process to be performed without exposing and potentially damaging the 2D material being processed. Finally, as an example relevant in terms of innovative device architectures, we present how the mild lithography approach can be used to achieve ordered arrangements of gold nanoparticles on 2D materials.

## Abstract 2

Mild lithography allows patterning of 2D materials while minimizing processing-induced defects. Thus, their structural integrity and intrinsic properties are preserved.

## Graphical abstract

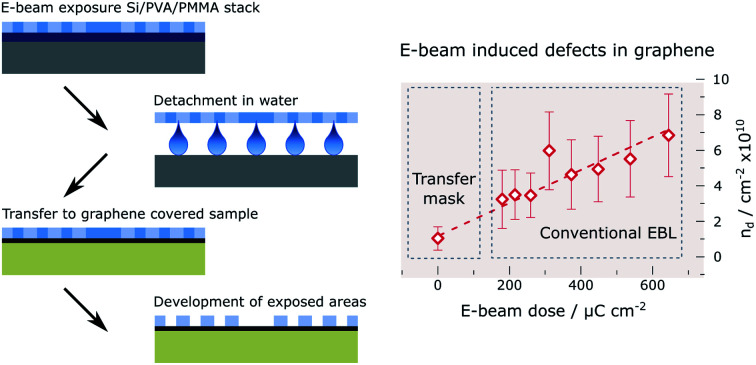

## Introduction

Today's device structures inherently become more complex as their functionalities steadily advance. The function of a device is a result of the interplay of its structure and its material constituents. Typically, the device performance is the better, the more ideal the realization of the device is. In particular, in the context of ‘More than Moore’, it is essential to find ways of incorporating novel materials, such as 2D materials, into the device structure in order to achieve more functionalities and to fully explore their potential. Lithographic techniques have become indispensable in this context.^1^ The ability to transfer patterns into sacrificial layers on top of the actual sample or device is a key process step in almost every bottom-up or top-down structuring process. In both cases, the structured sacrificial layer ensures that material is either deposited or removed selectively. This rather simple concept is still used today not only to fabricate electronic devices, like transistors, or to contact samples by the deposition of conducting tracks, but also in more sophisticated ways as in the case of selective area growth of nanowires.^2^ Common to all these techniques is that, in the structuring process of the sacrificial layer, either the chemical bonds of the resist are broken, or polymerization is induced locally, depending on whether a positive or negative tone resist is used. This makes it possible to dissolve the treated (untreated) areas of the resist in a suitable developer solution. In photolithography (PL) UV light provides the required energy, whereas in electron beam lithography (EBL) a focused electron beam is used to expose the resist. However, achieving a continuous improvement of the spatial resolution in the structuring process is mandatory in order to meet the requirements of advancing miniaturization, for this purpose, it is essential to increase the energy of photons, *i.e.* decrease their wavelength, according to Abbe's diffraction limit. For example, in the case of extreme UV lithography (EUVL), photons with an energy of almost 100 eV (13.5 nm) are employed today.^3^ Electron energies for a typical EBL process are in the range of several keV. Regardless of the different nature of the interaction between photons and electrons with matter, even significantly lower energies of the corresponding particles would already be sufficient not only to expose the resist but also to alter or damage the sample itself. The danger of inducing damage in the structuring process is especially high for the so-called 2D materials,^4–15^ which are extremely delicate due to their ultimate surface-to-volume ratio and the resulting strong dependence of their physical properties on the surface defect density.^16–20^ To address this issue, we present a method that allows us to structure the resist mask without exposing the delicate sample to UV light or an electron beam. Our method is based on a wet transfer of the patterned resist film with polyvinyl alcohol (PVA) as used similarly for transferring exfoliated 2D materials.^21,22^ Floating transfer in itself is not a new concept but is used in various applications based on different protocols, *e.g.* the transfer of photonic crystals^23,24^ and anodic aluminum oxide templates.^25^ However, to our knowledge it has not been used to transfer preexposed resist masks to structure easily damageable 2D materials as demonstrated here. In this study, we restricted ourselves to EBL and polymethyl methacrylate (PMMA) as the resist. However, this approach is more widely applicable, *e.g.*, it may be employed in PL processes or in combination with other resists as long as they are not soluble in water. In individual cases, chemical reactions between the PVA and the resist need to be considered.

## Experimental

### Preparation of mild lithography masks

**Fig. 1:**
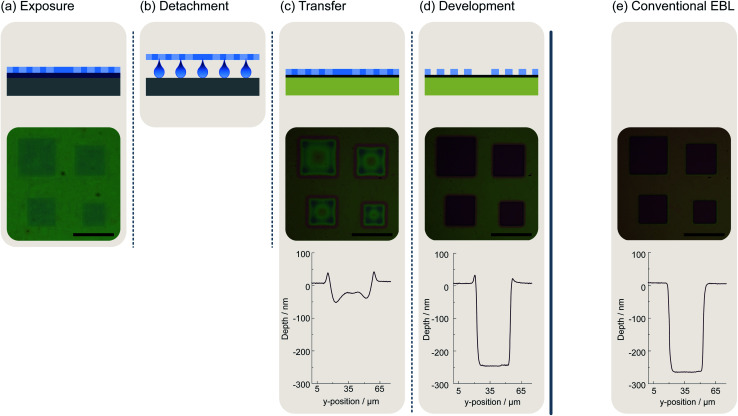
Illustration and evaluation of the mild lithography approach including mask preparation and the mask transfer process in comparison with the conventional EBL process for mask preparation. (a) The Si/PVA/PMMA (grey/darker blue/lighter blue) after being partially exposed (mid blue) by an electron beam. The exposed areas are already visible in the light microscopic image showing a test pattern consisting of squares of different sizes. The largest square in the upper left of the pattern has an edge length of 40 μm. (b) The PVA is dissolved in water leading to a separation of the PMMA mask and the silicon substrate. The PMMA mask floats on the water surface. (c) The floating resist mask can be picked up with the actual sample. Here, the orange box represents a Si/SiO_2_ substrate while the black layer corresponds to a two-dimensional material. The corresponding microscope image and profile show that the mask is already partially developed. The profile belongs to the upper left square with an edge length of 40 μm (d) the exposed areas of the resist are dissolved during the development the remainder defines the pattern on the sample surface. The corresponding microscope image and the profile belonging again to the upper left square reveal that the exposed PMMA is dissolved completely. At the edge of the structure exist minor elevations. (e) The light microscopic image and the profile of the same pattern in PMMA resist obtained by conventional EBL on a reference silicon wafer. The scale bars correspond to a length of 50 μm, respectively.

Fig. 1 shows the sequence of the individual process steps of our novel mild lithographic approach for patterning 2D materials by a mask transfer process. In a first process step, the desired mask pattern is prepared on a sacrificial substrate, a silicon wafer. After cleaning the silicon wafer in acetone and isopropyl alcohol (IPA) and a subsequent dehydration bake at 180 °C on a hot plate for 5 minutes, a layer of PVA and another of PMMA are successively deposited. To deposit the PVA film a solution of 2% PVA (PVA powder purchased from *SigmaAldrich*) dissolved in water is dispensed on the wafer by spin coating at 3000 RPM for 30 s followed by heat treatment on a hot plate at 100 °C for 30 s. The PMMA employed is produced by *Microchem*. It has a molecular weight of 950k and the solution contains 4% in anisole. Spin coating at 1500 RPM for 90 s leads to a film thickness of approximately 300 nm. The subsequent pre-bake step is performed on a hot plate at 120 °C for 90 s. At this step, it is important to deviate from the pre-bake temperature of 180 °C specified by *Microchem* on the datasheet, since we found that the solubility of PVA in water decreases when heat-treated at higher temperatures. For example, it takes several hours instead of a few minutes to remove the PMMA mask after it has been heated to 150 °C instead of 120 °C, and it is not possible at all to remove it after heating to 180 °C.

The substrate prepared in this fashion can be used in a standard EBL process (Fig. 1(a)). Typical parameters for the EBL process are an exposure dose of 200 μC cm^−2^ at a beam current of 200 pA and an acceleration voltage of 15 keV.

Before the undeveloped PMMA mask can be transferred to the substrate with the layer of interest, *e.g.*, the 2D material, it needs to be detached (Fig. 1(b)). For this purpose, it is essential to break off the edges of the Si/PVA/PMMA stack. This ensures that the PVA layer at the edges of the sample is not covered by PMMA and thus water can interact with the PVA. This can of course be done prior to the lithography step. For the actual transfer process, one needs to place the sample flat on a water surface using a pair of tweezers. The Si/PVA/PMMA stack will float on the water surface due to the surface tension. A meniscus forms at the edges of the sample, which leads to wetting of the PVA and results in the dissolution of the PVA film.

The PMMA mask can now be transferred to the actual sample (Fig. 1(c)). After a couple of minutes, the PMMA mask is detached from the substrate and floats on the water where it can be picked up with the substrate or thin-film sample to be structured.

Prior to developing the transferred PMMA mask, the sample needs to dry for about 30 minutes until the visible water film between the substrate and the PMMA is evaporated. Afterwards, the sample is further dried on a hot plate for 30 minutes at 150 °C to remove residual water. The mask can be developed afterwards and used as any other mask structured in a standard lithography process (Fig. 1(d)).

In addition to the schematic representations of the individual process steps, Fig. 1 also exemplarily shows microscope images and structural height profiles of a test structure. The test pattern comprises an arrangement of squares of different sizes. The largest square has an edge length of 40 μm. We deliberately chose a relatively large test structure to demonstrate that the PMMA mask remains stable even when large contiguous sections are exposed. Directly after the exposure, the squares are already visible since the electron beam causes a change of the refractive index in the exposed areas (Fig. 1(a)). After transferring the mask onto a Si/SiO_2_ wafer with an oxide thickness of 275 nm, both the microscope image and the height profile show that the mask is already partially developed. This is a special property of PMMA and would not necessarily occur with other resists (Fig. 1(c)). However, after the actual development of the structure (Fig. 1(d)), the microscope images and the height profiles show that the quality of the structures is not impaired by this behavior. They possess the same nominal size as a reference structure of the same pattern prepared by conventional EBL of PMMA on a Si/SiO_2_ substrate (Fig. 1(e)). The height profile exhibits only a small pile-up at the edge of the structure prepared by mask transfer.

### Validation methods

To further illustrate the usefulness of the mild lithography approach described above, we compare graphene layers onto which we have transferred a PMMA mask with corresponding graphene layers onto which we have directly written a pattern by conventional EBL. The samples studied were prepared using commercially available single-layer graphene (Easy Transfer Graphene by *Graphenea Inc.*) grown by chemical vapor deposition (CVD) on copper foil providing a coverage of >95%. Accordingly, it is polycrystalline with a flake size of about 20 μm and possesses few overgrown regions with more than one layer observable in the light microscope due enhanced contrast. Corresponding Raman spectra of multilayer sites are characterized by a blue shift of the 2D mode and can thus be easily excluded from the analysis.^26^ However, care was taken not to include any of these sites in the Raman mapping areas. The graphene has been transferred onto a wet oxidized silicon wafer (*Siegert Wafer*) with a nominal oxide thickness of 275 nm. The electron beam lithography processes are performed with a *JEOL JSM-7001F* scanning electron microscope equipped with a thermal field emission electron source and a *XENOS Semiconductor Technologies XeDraw 2* lithography stage. The acceleration voltage and the beam current are set to 15 keV and 200 pA, respectively. We varied the exposure dose systematically on two samples from different manufacturer batches and two separate sites of the samples were exposed for each dose to account for any imhomogeneities and batch to batch variation. To evaluate the impact of the e-beam exposure on the graphene, we recorded a Raman mapping consisting of 36 spectra for each site for averaging. These mappings are performed in back-scattering geometry with a setup consisting of a *Leica* top-illuminating bright-field microscope with a *Leica* 50×/0.75 NA objective and a *Renishaw InVia* spectrometer equipped with a charge-coupled device camera providing a spectral resolution of 1.5 cm^−1^. We use a frequency-doubled Nd:YAG laser emitting light with a wavelength of 532 nm. The applied laser power is 0.5 mW in all experiments.

## Results and discussion

### How lithographic procedures may damage graphene

**Fig. 2:**
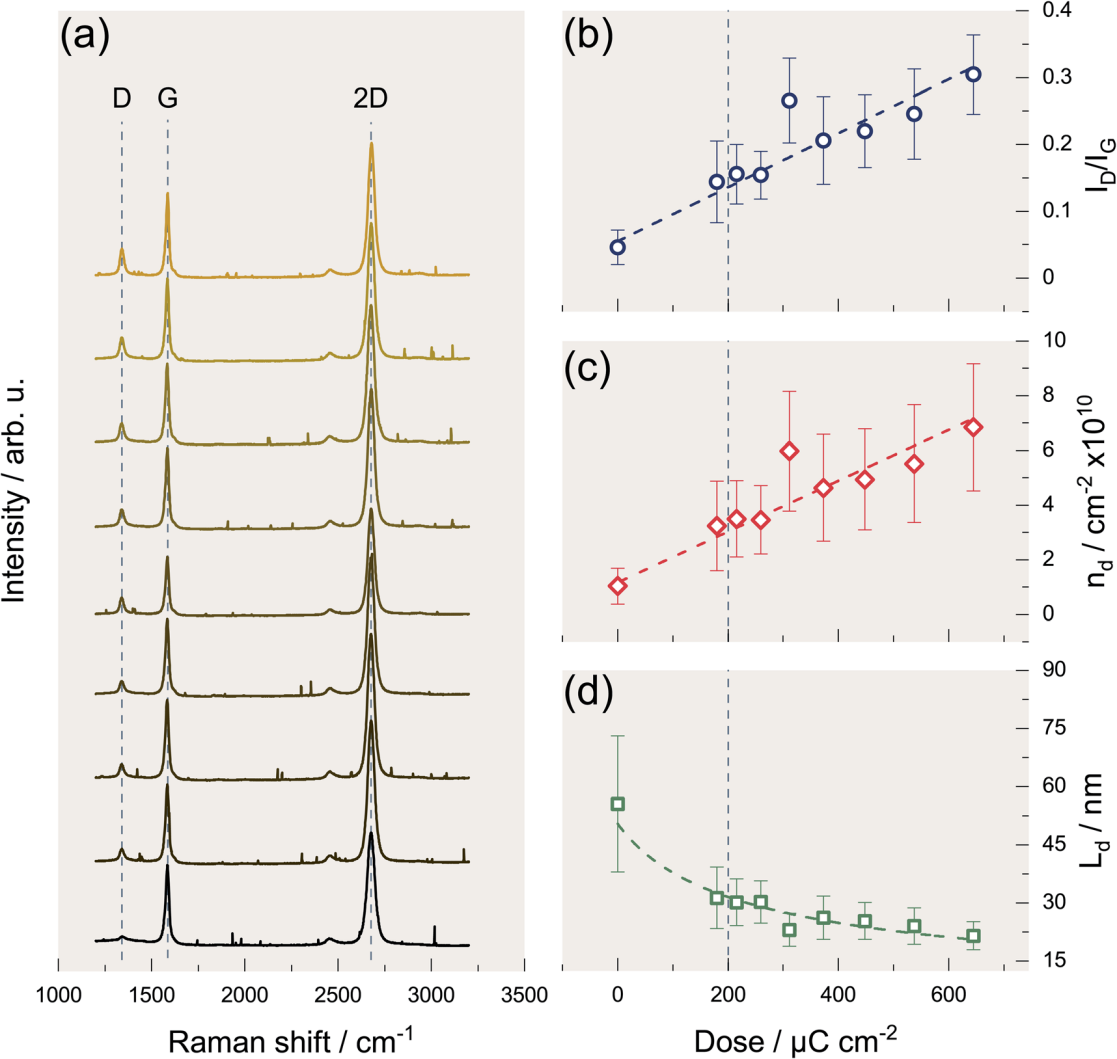
Increasing defect density of graphene with increasing electron beam exposure in an EBL process. (a) Accumulated Raman spectra of graphene for differently exposed areas displaying the typical D, G, and 2D mode. Each Raman spectrum is obtained by the accumulation of 36 individual Raman spectra measured by a Raman mapping of the exposed area. The exposure dose increases from the bottom (0 μC cm^−2^) to the top (650 μC cm^−2^) indicated by the color gradient. The intensity of the defect-induced D mode increases with increasing exposure dose. The randomly distributed narrow peaks correspond to cosmic rays. (b) Dependence of the intensity ratio of the D and G mode of graphene on the exposure dose fitted by a linear function. The *I*_D_/*I*_G_ ratios are obtained by averaging over the 36 spectra of each mapping. The error bars correspond to the corresponding standard deviation. The dashed vertical line corresponds to the exposure dose used in a standard EBL process (c) calculated values of the defect density according to Cançado *et al.* using the *I*_D_/*I*_G_ ratios depicted in (b). The error bars are obtained by combining the uncertainty value given by Cançado *et al.* and the standard deviation obtained in (b) using error propagation of independent variables. (d) The resulting mean distance of two defects calculated from the defect density (c). The error bars are again obtained by combining the uncertainty value given by Cançado *et al.* and the standard deviation obtained in (b) using error propagation of independent variables.

Fig. 2(a) shows the accumulated Raman spectra of the areas on graphene exposed to different e-beam doses. The spectra are stacked according to exposure dose which increases from bottom to top. The lowest spectrum belongs to a graphene sample onto which we transferred the mask and thus belongs to a dose of 0 μC cm^−2^. For the spectra above, the dose increases successively from 180 to 650 μC cm^−2^. Note a typical dose for structuring PMMA by EBL is 220 μC cm^−2^. The Raman spectra reveal the modes typical of graphene. The so-called G mode belongs to the zone center phonon and has E_2g_ symmetry. The 2D mode results from a resonant two-phonon process involving the excitation of two phonons with A_1_-symmetry near two *K*/*K*′ points with opposite wave vectors. In the Raman process leading to the D mode, a phonon with A_1g_ symmetry is excited near a *K*/*K*′ point. However, to satisfy the conservation of momentum, the excited electron undergoes an additional elastic scattering with a defect. This makes the D mode only observable in defected graphene. Therefore, the relative intensity of the D mode provides a measure for the defect density of the graphene sample under observation.^27^Fig. 2(b) depicts the D mode intensity normalized to the intensity of the G mode. Fig. 2(b) shows that this intensity ratio and hence the defect density of the graphene sample increases linearly with increasing exposure dose. This finding indicates that the e-beam in the dose range under study always causes the same type of defect, as anticipated and is significant for the typical dose of 200 μC cm^−2^ (as indicated by the vertical dashed lines in the graphs (b) to (d)). Furthermore, the density of point-like defects increases by about one order of magnitude when graphene is exposed to an e-beam dose of 650 μC cm^−2^.

A quantitative interpretation in terms of absolute numbers for the defect densities caused is difficult because the Raman cross-section for the D process not only depends on the density of defects but also on the kind of defect.^28–31^ However, in the following we will assume that the defects caused by e-beam irradiation are similar to those caused by Ar ion bombardment in terms of the D process Raman cross-section. According to Cançado *et al.*, it is then possible to convert the *I*_D_/*I*_G_ ratio into a defect density.^32^ The result is depicted in Fig. 2(c). Finally, Fig. 2(d) shows the corresponding average distance between two defects. The values derived are 4 × 10^10^ cm^−2^ and about 30 nm for the layer density of defects and the mean distance between defects, respectively, for an electron dose typical for a conventional EBL. For the highest dosage 650 μC cm^−2^ the defect density linearly increases to roughly 4 × 10^10^ cm^−2^, while the average defect distance decreases to about 20 nm. These numbers are quantitatively comparable for both graphene samples prepared and demonstrates that the formation of defects, which is inevitable in a conventional EBL process, may considerably affect the device performance.

Within this study, we cannot determine the exact nature of the defects present. Essentially, there are two plausible types. The first would be the transition of graphene into sp^2^ nanocrystallites due to the electron bombardment.^10–12,33,34^ It has been shown that this effect already occurs at irradiation with photons of the energy of 2.5 eV (488 nm)^7^ and that this is possibly related to the formation of Stone–Wales (SW) defects.^35,36^ From DFT calculations it is known that the formation energy of this type of defect is about 4.6 eV (ref. 37) so that the defects form either thermodynamically by heating or directly *via* two-photon absorption. In either case, this type of defect would explain the bulging of the irradiated regions that is also observed.^35,36^ Furthermore, due to already existing defects and the weaker bonds in their direct vicinity, the necessary energy for the generation of additional defects may be lowered.^38^ The second possible type would be due to the partial hydrogenation of graphene in the EBL process, which also leads to the emergence of an intense D mode.^5,8,9,39^ The hydrogen necessary may be provided by the PMMA in this case. In contrast, the knock-on displacement of carbon atoms can be excluded as a possible cause of defect formation. The energy of the electrons with 15 keV is simply not sufficient to cause this type of displacement, since acceleration voltages of at least 80 kV are necessary to transfer the required energy.^40–42^ Corresponding processes thus have only been observed and even exploited in transmission electron microscopy (TEM).^43^

**Fig. 3:**
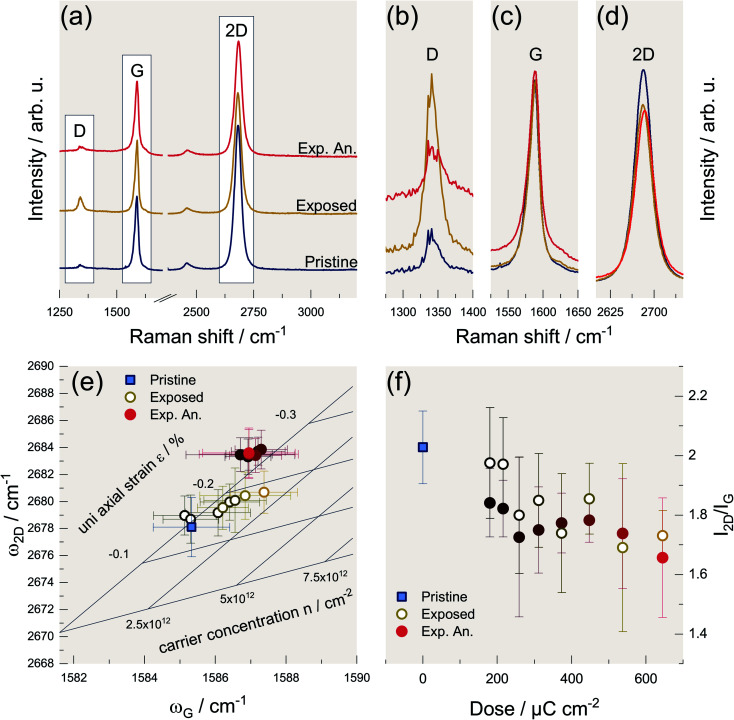
Evaluation of vacuum heating concerning its capabilities for restoring the properties of graphene after electron irradiation. (a) Stacked Raman spectra of a pristine graphene sample (blue), the graphene sample was exposed to an electron dose of 650 μC cm^−2^ (yellow), and spectra of the same irradiated graphene sample after annealing it in a vacuum furnace at 1 × 10^6^ mbar and 300 °C for 30 minutes (red). (b)–(d) close-ups of the D, G, and 2D mode, respectively, of the three Raman spectra shown in (a). The spectra are not stacked so that the intensities can be compared directly. (e) Vector decomposition of strain *ε* and carrier density *n* according to Lee *et al.* by plotting the G mode frequency *versus* the 2D mode frequency. The data of the pristine sample (blue square) serves as a reference. The data of the exposed graphene samples are represented by rings, the color gradient indicates an increasing electron dose (see (f)). The filled circles represent data of the same exposed graphene samples after vacuum annealing. The error bars correspond to the standard deviation. (f) Dependence of the *I*_2D_/*I*_G_ ratio on the deposited electron dose. Again, data of the pristine graphene sample (blue square) serves as a reference and the rings represent data of the samples after exposure while the circles represent data of the samples after vacuum annealing.

Regardless of the precise origin of the defects, we observe that the intensity of the D mode can be reduced to its original value by vacuum annealing as described in the literature.^5,9,11^ Accordingly, we annealed the samples evaluated in Fig. 2 at 300 °C and 1 × 10^−6^ mbar for 30 min as well, to be able to compare our results with the literature. However, due to the low pressure, the cooling-down process takes several hours, making it virtually impossible to reliably study the time dependence of the annealing procedure. However, in the literature, exposed graphene was also annealed at 600 °C for 2 h in Ar/H_2_ and qualitatively comparable results were obtained.^44^ The results are exemplarily shown in Fig. 3(a and b) for the highest dose of 650 μC cm^−2^. The individual comparison of the G and 2D modes is shown in Fig. 3(c and d), respectively. It can be seen that, as expected, the intensity of the G mode is hardly affected. However, the 2D mode loses intensity due to the additional defects.^28,45,46^ The latter cannot be reversed by the temperature treatment indicating that annealing does not lead to a full recovery of graphene's structural integrity, although the D mode intensity approaches the initial value of the pristine material.

This behavior and the spectral position of the modes will be discussed in more detail in the following. Lee *et al.*^47^ introduced a convenient way to study graphene in terms of strain and charge doping solely based on Raman spectra, *i.e.*, the phonon frequencies of G and 2D mode. We applied this method to our data and the result is shown in Fig. 3(e). By introducing the oblique coordinate system, it is possible to read off directly the uniaxial strain and the charge carrier density (hole doping). This representation would imply that the graphene, at low electron doses, is initially compressively strained and becomes additionally p-doped at higher doses. This p-doping can be explained by a charge accumulation within the substrate due to the electron beam^48^ and by the activation of additional adsorption sites leading to an enhanced p-doping due to atmospheric oxygen.^49,50^ However, since the corresponding representation cannot unambiguously distinguish between electron and hole doping, electron doping of the graphene would be likewise possible, as was observed by Childres *et al.*^51^ Another effect that would also lead to a stiffening of the G mode is the softening of the *k* selection rule caused by phonon confinement.^28,34^ However, the phonon confinement should also lead to an increase of the G mode's full width at half maximum (FWHM) *Γ*_G_. As we observe rather a decrease of *Γ*_G_ with increasing electron dose, we assume that charge is indeed the underlying cause of the blue shift of the G mode.^52^ The reason for the observed compressive strain is not obvious and its determination is beyond the scope of this work. Since other groups observe tensile strains due to electron irradiation,^12,15^ it is reasonable to assume that this correlation is not universal and depends on the individual specimen or preparation conditions.

After annealing, the picture changes as there is no longer a noticeable distinction between the different specimens in terms of strain and charge carrier density. However, the compressive strain increases significantly during annealing. This is associated with the formation of ripples in graphene caused by the difference in thermal expansion coefficients of graphene and SiO_2_.^44,47,50,53^Fig. 3(f) depicts the dependence of the intensity ratio of the G and 2D mode *I*_2D_/*I*_G_ on the electron dose. A decrease in this ratio is indicative for the presence of defects that do not contribute to the D mode process. These are, for example, strain, charged impurities, and also zigzag edges.^27,54^ This shows that the original state of the graphene cannot be entirely restored by annealing, which was also found by other authors. For example, He *et al.* reported the absence of the Quantum Hall effect in comparable samples.^49^ We can conclude that a thermal annealing step should be avoided in a device process involving supported graphene as it will alter its properties.

### Mild lithography for systematic nanoparticle arrangements

**Fig. 4:**
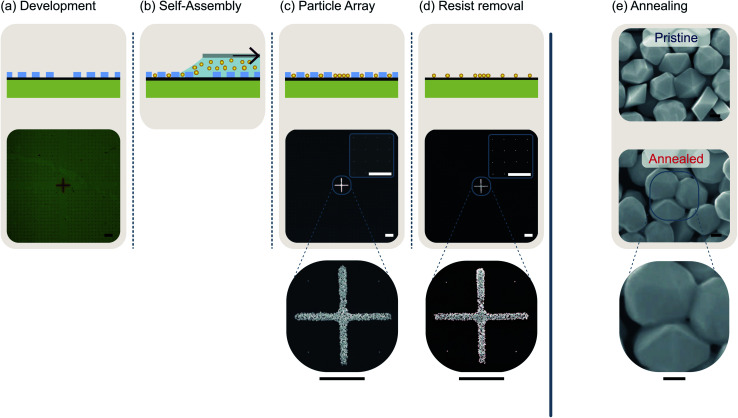
Illustration and evaluation of the systematic arrangement of nanoparticles by template-assisted self-assembly using patterns defined by mild lithographic processing. (a) The starting point for the arrangement of nanoparticles is a resist mask with suitable particle traps. The microscope image shows a section of a PMMA mask on a Si/SiO_2_ wafer with square particle traps with an edge length of 300 nm. Since particles with a diameter of 250 nm are to be arranged, only one particle fits into each particle trap. The cross in the center serves as a marker to find the particle arrays on the sample. (b) The structured particle traps are filled with gold nanoparticles (yellow circles) in a self-assembled alignment. The cavity between the PMMA layer and a glass slide (light grey), which is slowly retracted, is filled with a suspension of gold nanoparticles. The emerging meniscus exerts a force on the particles, which pushes them into the resist gaps. (c) Ideally, this leads to a situation where each cavity is filled with one gold nanoparticle and the marker crosses are densely packed with gold nanoparticles. The scanning electron microscopy (SEM) image shows that this is indeed the case. The close-ups confirm that the cavities are each filled with individual gold nanoparticles as well as that the cross is densely packed with gold nanoparticles. (d) After dissolving the PMMA mask in acetone, properly arranged nanoparticles are obtained. This is shown by the SEM images. (e) Effect of vacuum annealing on 250 nm gold nanoparticle arrangements. The upper SEM image shows the center of a cross-shaped mark as transferred. The lower SEM image shows a comparable mark after vacuum annealing at 300 °C. One observes that the particles' facets blur and that adjacent particles are sintered together. The scale bars in (a)–(d) correspond to a length of 10 μm and the scale bars in (e) correspond to a length of 300 nm, respectively.

Here, we present a possible application of the masks in a process step that may be used in device fabrication. We will demonstrate how the masks can be used to achieve ordered arrangements of isolated gold nanoparticles by template-assisted self-assembly on graphene.^55,56^ Template-assisted self-assembly has also been successfully employed to create resonator structures consisting of a few gold nanoparticles which exhibit high Raman enhancement factors due to gap modes.^57^ Especially in combination with 2D materials, plasmonic nanoparticles are often used to enhance the interaction with light and generate an additional photocurrent.^58–61^ The assembly process is illustrated in Fig. 4. The starting point is a transferred mask with cylindrical particle traps whose diameters are slightly larger than that of the particles to be arranged combined with larger cavities that serve as finder marks (Fig. 4(a)). Here, we want to arrange nanoparticles with an average diameter of 250 nm. Therefore, we design the particle traps as cylinders with a diameter of 300 nm and choose the PMMA to have an approx. height of 300 nm as well. Of course, almost any other particle arrangement can be realized by a skillful choice of the trap geometry.^62^ A certain amount of a suspension containing nanoparticles in an aqueous solution is deposited into the narrow gap between the sample and a glass slide. This glass slide is slowly retracted along the sample surface using a stepper motor at a speed of a few millimeters per hour. A meniscus is formed that applies a force with a component normal to the sample plane pushing the nanoparticles into the dedicated cavities (Fig. 4(b)). This ideally leads to the situation shown in Fig. 4(c) where every cavity is filled with a tightly packed monolayer of nanoparticles. These particles are rigidly attached to the substrate allowing the dissolution of the PMMA mask in acetone without being washed away (Fig. 4(d)). Finally, Fig. 4(e) impressively illustrates that a subsequent annealing procedure aimed at healing defects in the graphene is not possible without affecting the gold nanoparticles because 300 °C are already sufficient to sinter adjacent gold nanoparticles together and causing gaps between the particles to disappear. However, it is precisely these gaps which act as resonators and result in the desired enhancement of the light field in the context of plasmonic devices.^57^ Plasmonic nanostructures with resonators of gold nanoparticles can be used to maximize the achievable photo current.^63^ Thus, destroying the corresponding gaps between gold nanoparticles, one squanders immense potential in terms of device efficiency.

## Conclusions

In summary, the integration of 2D materials into devices offers the extraordinary potential to significantly shape the technology of tomorrow beyond ‘Moore’. However, the extent to which this is possible in practice will depend on the availability of processing methods that allow 2D materials to be integrated into devices whilst maintaining their high pristine quality and structural integrity. Established methods such as conventional EBL do not meet these requirements, as they are likely to induce defects in 2D materials due to the high-energy radiation required in the lithographic process. Such defects degrade the 2D material and have a major impact on its properties. Subsequent recovery of these initial properties by thermal annealing is possible only to a limited extent. Thus, it is essential to develop mild processing methods in order to be able to integrate 2D materials into devices retaining their highest possible quality. We propose a mild lithography process which remedies these issues. Our approach aims at complementing traditional processing methods by an additional transfer step. This allows the mask writing process to be performed at equivalent quality without exposing and, thus, potentially damaging the 2D material being processed. The limiting factor of the method is basically the writing speed of the EBL process. Apart from that, complete wafers can be processed by mask transfer. In this respect, we see the limitation of scalability essentially in the piece-by-piece processing. In addition to the use in processing 2D materials, our approach may turn out useful in many other fields of application. For example, the transfer of a mask offers the possibility to effortlessly structure a curved surface or a non-conductive substrate.

## Data availability

The data that support the findings of this study are openly available in JLUpub at http://dx.doi.org/10.22029/jlupub-169.

## Author contributions

Marcel Weinhold: conceptualization, formal analysis, investigation, methodology, resources, validation, visualization, writing – original draft. Peter J. Klar: conceptualization, methodology, writing-review & editing, supervision.

## Conflicts of interest

There are no conflicts to declare.

## Supplementary Material
